# Changes in the hemagglutinin of H5N1 viruses during human infection – Influence on receptor binding^☆^

**DOI:** 10.1016/j.virol.2013.08.010

**Published:** 2013-12

**Authors:** Martin Crusat, Junfeng Liu, Angelina S. Palma, Robert A. Childs, Yan Liu, Stephen A. Wharton, Yi Pu Lin, Peter J. Coombs, Stephen R. Martin, Mikhail Matrosovich, Zi Chen, David J. Stevens, Vo Minh Hien, Tran Tan Thanh, Le Nguyen Truc Nhu, Lam Anh Nguyet, Do Quang Ha, H.Rogier van Doorn, Tran Tinh Hien, Harald S. Conradt, Makoto Kiso, Steve J. Gamblin, Wengang Chai, John J. Skehel, Alan J. Hay, Jeremy Farrar, Menno D. de Jong, Ten Feizi

**Affiliations:** a Oxford University Clinical Research Unit, Hospital for Tropical Diseases, Ho Chi Minh City, Vietnam; b Department of Medical Microbiology, Academic Medical Center, University of Amsterdam, Amsterdam, The Netherlands; c MRC National Institute for Medical Research, London, United Kingdom; d The Glycosciences Laboratory, Department of Medicine, Imperial College London, United Kingdom; e REQUIMTE/CQFB, Faculty of Science and Technology, New University of Lisbon, Caparica, Portugal; f Institute of Virology, Philipps University, Marburg, Germany; g Hospital for Tropical Diseases, Ho Chi Minh City, Vietnam; h GlycoThera GmbH, Hannover, Germany; i Department of Applied Bio-organic Chemistry, Gifu University, Japan; j National University of Singapore, Singapore

**Keywords:** H5N1 influenza infection, Pyrosequencing, Hemagglutinin, Receptor specificity, Hemagglutination assays, Receptor binding, Carbohydrate microarray, Biolayer interferometry, Synthetic sialylglycopolymers, Hemagglutinin X-ray crystal structure

## Abstract

As avian influenza A(H5N1) viruses continue to circulate in Asia and Africa, global concerns of an imminent pandemic persist. Recent experimental studies suggest that efficient transmission between humans of current H5N1 viruses only requires a few genetic changes. An essential step is alteration of the virus hemagglutinin from preferential binding to avian receptors for the recognition of human receptors present in the upper airway. We have identified receptor-binding changes which emerged during H5N1 infection of humans, due to single amino acid substitutions, Ala134Val and Ile151Phe, in the hemagglutinin. Detailed biological, receptor-binding, and structural analyses revealed reduced binding of the mutated viruses to avian-like receptors, but without commensurate increased binding to the human-like receptors investigated, possibly reflecting a receptor-binding phenotype intermediate in adaptation to more human-like characteristics. These observations emphasize that evolution in nature of avian H5N1 viruses to efficient binding of human receptors is a complex multistep process.

## Highlights

• Changes in receptor binding of HA during H5N1 human infection were identified.
• Single A134V and L151F substitutions caused reduced affinity for avian receptors.
• Glycan array analyses were used to identify changes in receptor binding specificity.
• Structural basis for altered receptor binding was examined by X-ray crystallography.

## Introduction

Since 1997, highly pathogenic avian influenza A(H5N1) viruses have spread among poultry and wild birds in Asia, the Middle-East, Europe and Africa and caused over 600 reported human infections in 15 countries with a case-fatality ratio of approximately 60% (WHO, 2012). Sporadic human infections continue to occur in countries where A(H5N1) viruses have become endemic in birds, providing a persistent threat to global health due to the possibility of virus adaptation towards efficient transmission among humans and ensuing pandemic spread. Recent evidence from experiments with ferrets, a widely accepted animal model for influenza in humans, suggest that only a limited number of genetic changes is needed for airborne transmission of H5N1 viruses (Imai et al., 2012; Herfst et al., 2012). Thus, monitoring of genetic changes, especially during human infections, and studying the relevance of such changes for human adaptation remains essential.

The molecular mechanisms that allow avian influenza viruses to cross the species barrier from birds to humans are incompletely understood. However, a prerequisite for efficient transmission of avian viruses between humans is a change from preferential recognition by the virus hemagglutinin (HA) of α2-3-sialyl-galactose-terminating host cell receptors, predominant in avian respiratory and gastrointestinal epithelia, to those terminating in α2-6-sialyl-galactose, which predominate in the human upper respiratory tract (Rogers and Paulson, 1983; Rogers and D'Souza, 1989). Accordingly, HAs of the 20th century pandemic viruses (H1N1 in 1918, H2N2 in 1957 and H3N2 in 1968) evolved from their original recognition of avian α2-3-sialyl- to preferential α2-6-sialyl-receptor binding (Connor et al., 1994; Matrosovich et al., 2000; Glaser et al., 2005; Stevens et al., 2006b; Tumpey et al., 2007). Thus to better understand the nature and significance of such adaptive changes, it is essential to monitor HA changes that may affect receptor specificity of avian influenza viruses, particularly during human infection.

In this study, we screened receptor-binding preferences of thirty H5N1 viruses, isolated in MDCK cells, from poultry and humans in Vietnam during 2004 and 2005, by comparing hemagglutination patterns using horse and guinea pig red blood cells (RBCs), which differ in sialic acid receptor distribution (Ito et al., 1997b; Medeiros et al., 2001). We detected patterns indicative of changes in receptor specificity in three human H5N1 isolates. Our analyses of these viruses and their egg-passaged counterparts indicated a rapid emergence of adaptive receptor-binding variants of H5N1 virus, and also demonstrated that marked discrepancies can occur in quasispecies distributions between clinical specimens and cell culture- or egg-grown viruses, thus emphasizing the need for genetic monitoring directly in clinical specimens.

## Results

### Agglutination of horse RBCs

To screen for the receptor-binding preferences of avian and human influenza H5N1 viruses isolated in MDCK cells, we compared the relative agglutination of horse RBCs, which express predominantly α2-3-sialyl sequences (Ito et al., 1997a) and guinea pig RBCs, which express both α2-3- and α2-6-sialyl sequences (Medeiros et al., 2001). As predicted, seasonal human H1N1 and H3N2 influenza A viruses (*n*=10) did not agglutinate horse RBCs, whereas avian influenza viruses (*n*=15; H5N1, H4N6, H6N1) isolated from poultry agglutinated efficiently both horse and guinea pig RBCs (Table 1). Out of 11 influenza H5N1 viruses isolated from upper respiratory tract specimens of humans, eight agglutinated efficiently both types of RBCs, similar to poultry viruses. However, two of the human H5N1 virus isolates (A/Vietnam/CL1/2004 and A/Vietnam/CL105/2005) resembled seasonal human influenza A viruses and did not agglutinate horse RBCs (Table 1). A third isolate (A/Vietnam/CL2009C/2005) showed a 15-fold lower hemagglutination titer with horse RBCs than with guinea pig RBCs (Table 1 and Supplementary Table S1). These results suggested that three of the 11 human H5N1 viruses had reduced binding to α2-3-sialyl receptors.

We investigated whether the hemagglutination properties of these three human H5N1 viruses would ‘revert’ to efficient horse RBC agglutination when replicating in the presence of predominantly α2-3-sialyl receptors. Indeed, after passaging the viruses five times in the allantois of embryonated chicken eggs, which contain only α2-3-sialyl receptors (Ito et al., 1997b), all three viruses (re)gained efficient agglutination of horse RBCs (Table 1). Passage in eggs did not, however, alter the agglutination patterns of other avian influenza viruses isolated from humans or poultry or seasonal human influenza viruses (Table 1). Plaque assays showed that the MDCK cell isolate and egg passaged variant of A/Vietnam/CL1/2004 and A/Vietnam/CL105/2005 exhibited similar growth characteristics in MDCK cells.

### Amino acid changes in HA

To identify amino acid changes in HA associated with the hemagglutination patterns of A/Vietnam/CL1/2004, A/Vietnam/CL105/2005, and A/Vietnam/CL2009C/2005, the sequences of HA1 of the MDCK cell isolates were compared with those following passage in eggs. A single amino acid difference was observed in the HA of each egg-grown virus (Table 2): Asp186Glu in A/Vietnam/CL1/2004, Val134Ala in A/Vietnam/CL105/2005, and Phe151Leu in A/Vietnam/CL2009C/2005 (H5 numbering). Changes in the first two viruses represented reversions to the avian H5 consensus sequence following egg passage. The consensus residue at position 151 is Ile rather than Leu. Interestingly, sequence analysis in the original clinical specimen revealed a subpopulation of 151Ile and not Leu (see below).

### Quasispecies distributions in clinical specimens and virus isolates

To determine whether the sequences in the MDCK cell isolates were representative of those present during the human infection and to analyze quasispecies populations, pyrosequencing specifically targeting HA positions 134, 186 and 151 was performed directly on clinical specimens as well as on the viruses cultured in MDCK cells and eggs (Table 2). These analyses confirmed the presence of Asp186 in the MDCK cell isolate of A/Vietnam/CL1/2004 and the reversion to Glu186 after egg passage. Surprisingly, direct pyrosequencing of viruses in clinical specimens from the nose and throat of the patient showed the presence of only Glu186, indicating that the Asp186 variant had emerged during passage in MDCK cells, but not during infection of respiratory epithelium of the patient.

Pyrosequencing also confirmed the predominance of the Phe151 variant(s) of A/Vietnam/CL2009C/2005 in the MDCK cell isolate which changed to Leu151 after egg passage. A throat specimen obtained from the patient, after 7 days of illness, contained 65% of the Phe151 variant, which increased to 100% in a sequential sample taken at day 8, at the time the specimen was taken for virus isolation in MDCK cells. Interestingly, the remaining 35% of virus quasispecies in the first specimen contained Ile at position 151, which represents the consensus residue at this position of the avian H5 sequence, and not Leu as observed in egg-passaged virus. Of note, a virus isolated in MDCK cells from a respiratory swab 5 days after the onset of illness in the same patient (A/Vietnam/CL2009/2005) had the consensus sequence with Ile151 (GenBank accession number DQ497729) (Tran et al., 2004; Beigel et al., 2005; de Jong et al., 2005b, 2006), indicating that the major virus population during earlier stages of infection had not mutated.

The MDCK cell isolate A/Vietnam/CL105/2005 consisted of a mixed virus population with similar proportions of Ala134 and Val134 variants, whereas the egg-cultured virus contained predominantly the Ala134 variant. In the original clinical specimen, obtained after 4 days of illness, Val134 variants predominated in the virus population at 74%.

### Recombinant viruses

To compare the effects of the observed amino acid changes on receptor binding, two sets of recombinant viruses, which contain an H5 gene together with seven PR8 genes, were constructed using reverse genetics (Table 3). In one set (rVN1194-like) single amino acid substitutions, corresponding to the residues altered during egg passage, were introduced into the HA of the parental ‘wild type’ recombinant A/Vietnam/1194/2004 virus (NIBRG14, rVN1194). In the second set of viruses additional substitutions were introduced, by mutagenesis or chemical synthesis, such that the HA sequences were identical to those of the corresponding MDCK cell isolates (Table 3). The agglutination patterns of the recombinant viruses with Val134 or Asp186 were comparable to those of the corresponding MDCK cell isolates (Tables 1 and 3). Attempts to recover viruses possessing Phe151 in HA, in a PR8 genetic background, for receptor binding studies were unsuccessful as passage in MDCK (as well as MDCK-SIAT1) cells resulted in mixed Phe151/Leu151 populations, whereas Leu151 was selected during passage in eggs. For crystallographic studies, HA of the Phe151 variant was produced using a vaccinia virus construct.

### Binding to soluble sialylglycopolymers

**Fig. 1:**
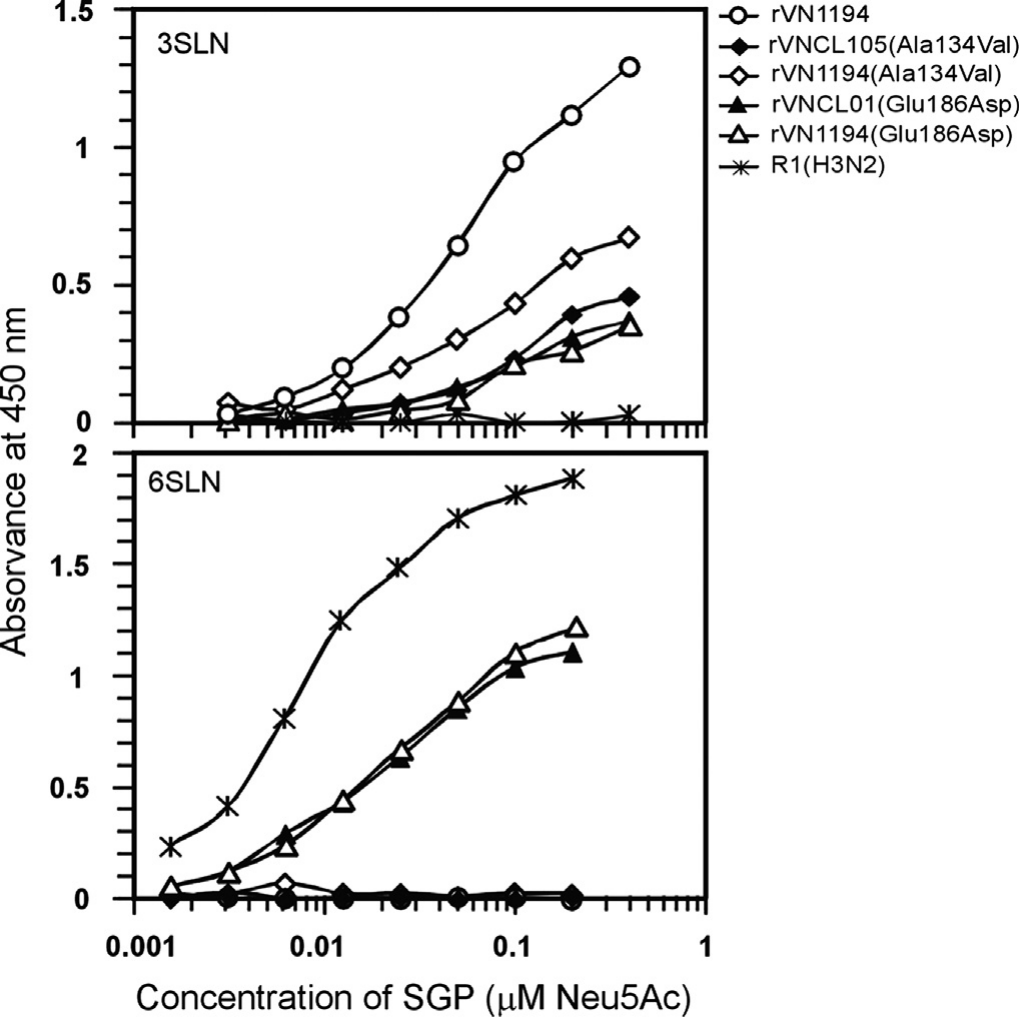
Binding of biotinylated sialylglycopolymers 3SLN-PAA (30 kDa) and 6SLN-PAA (1 MDa) to viruses adsorbed in 96-well microplates. H5N1 viruses rVN1194 (circles), rVN1194(Ala134Val) (diamonds), rVNCL105(Ala134Val) (filled diamonds), rVN1194(Glu186Asp) (triangles), rVNCL01(Glu186Asp) (filled triangles). Control human H3N2 virus R1 (asterisks). The data show averaged values of two replicates and are representative of two independent experiments performed on different days.

The effects of the mutations were analyzed by assaying the binding of soluble sialylglycopolymers (SGPs) to immobilized viruses. Initially, the low molecular mass (30 kDa) SGPs, α2-3-sialyl-N-acetyllactosamine (3SLN)-PAA and α2-6-sialyl-*N*-acetyllactosamine (6SLN)-PAA were used. There was strong binding of the 3SLN-PAA to the ‘wild type’ rVN1194, whereas binding to the four recombinant H5N1 viruses with mutations at positions 134 or 186 was significantly less avid. There was no detectable binding of this SGP to a human H3N2 virus R1, used as a control (Fig. 1). The low molecular mass 6SLN-PAA gave no binding to any of the H5N1 viruses, although there was binding to the human H3N2 virus R1 (data not shown). To increase the sensitivity of detection of binding of the viruses to the α2-6-sialyl sequence, we used the high molecular mass (1 MDa) 6SLN-PAA that has higher glycan valency. This SGP bound to the two recombinant viruses with a Glu186Asp mutation, although significantly less strongly than to the control human H3N2 virus R1 (Fig. 1); ‘wild type’ rVN1194 and the two recombinant Ala134Val mutants were not bound.

### Analysis of binding specificity by biolayer interferometry

**Fig. 2:**
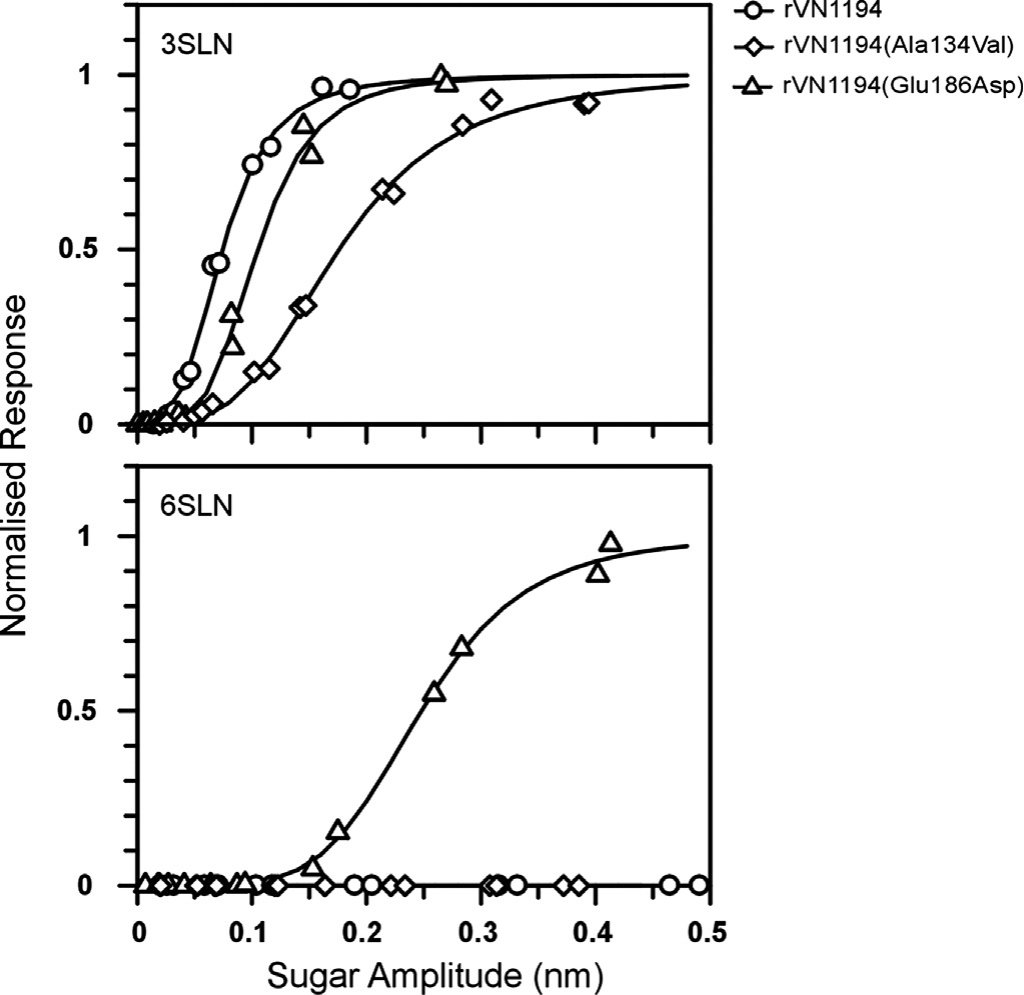
Virus binding analysis by biolayer interferometry. H5 viruses rVN1194 (circles), rVN1194(Glu186Asp) (triangles) and rVN1194(Ala134Val) (diamonds) were analyzed for binding to immobilized biotinylated 30 kDA sialylglycopolymers 3SLN-PAA and 6SLN-PAA.

Receptor binding of the recombinant H5N1 viruses rVN1194, rVN1194(Glu186Asp) and rVN1194(Ala134Val) was analyzed by biolayer interferometry using the biotinylated SGPs 3SLN-PAA and 6SLN-PAA immobilized on streptavidin-coated biosensors. Equilibrium binding measurements were recorded at varying sugar immobilization densities at a constant virus concentration. The three viruses showed binding to 3SLN (Fig. 2). In this assay format, the rVN1194(Glu186Asp) mutant virus bound with a slightly lower affinity (approximately 5-fold lower) to the 3SLN than did the ‘wild type’ rVN1194 virus, whereas the rVN1194(Ala134Val) mutant virus bound with much lower (approximately 20-fold) affinity. The rVN1194(Ala134Val) mutant, like the rVN1194 virus, showed no detectable binding to 6SLN. In contrast, the rVN1194(Glu186Asp) mutant showed detectable binding to 6SLN; this was, however, approximately 50-fold weaker than to the 3SLN.

### Patterns of oligosaccharide binding in carbohydrate microarray analyses

**Fig. 3:**
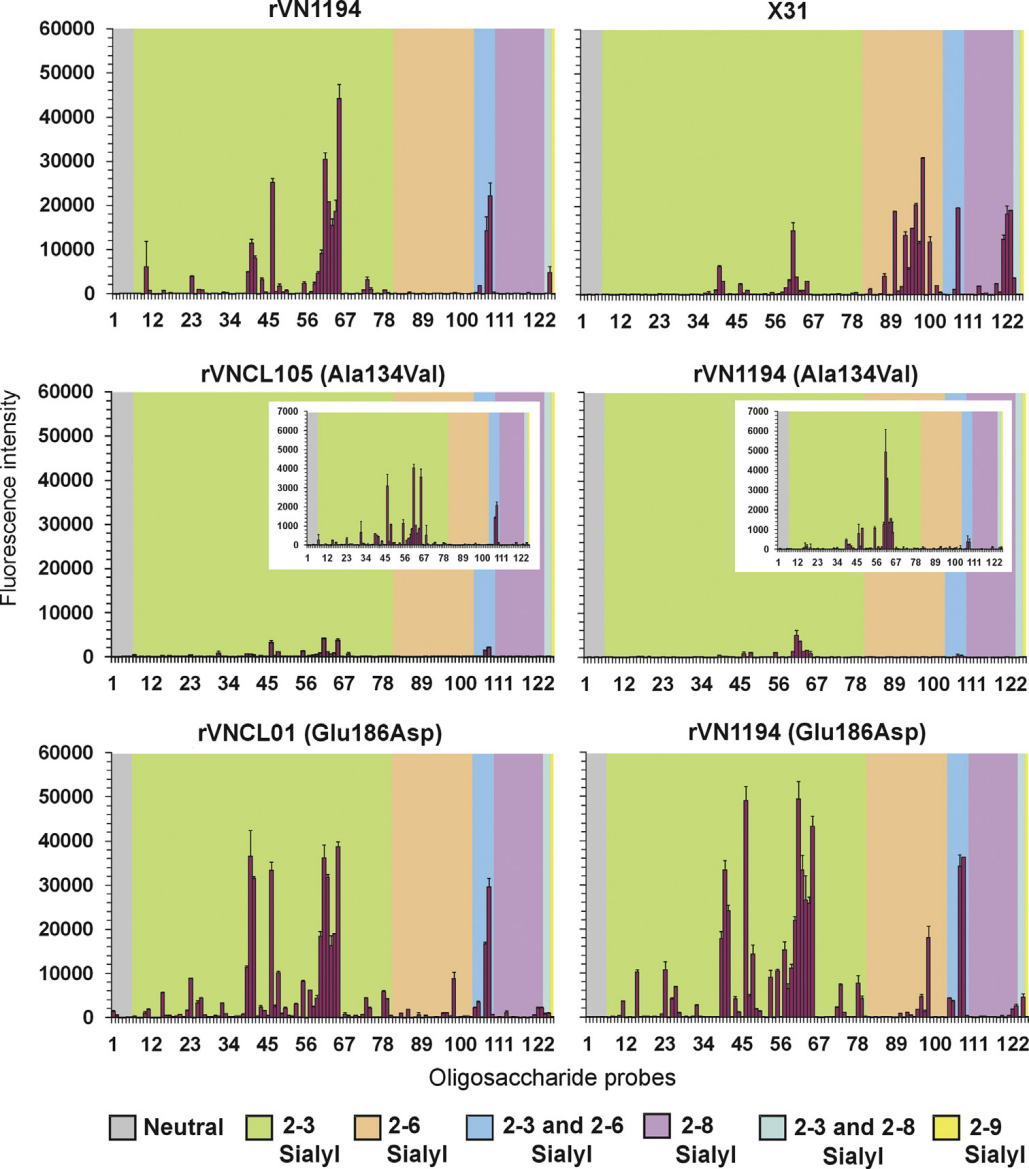
Carbohydrate microarray analyses of wild type and mutant H5N1 viruses and X31(H3N2) as control. Numerical scores for the binding signals are shown as means of duplicate spots at 5 fmol/spot (with error bars) and are representative of at least three independent experiments. The insets show enlarged the *Y*-axis scales for the 134 mutant viruses, which gave very low binding signals. The microarrays consisted of lipid-linked oligosaccharide probes, printed on nitrocellulose-coated glass slides. These are listed in Supplementary Table S2 and arranged according to sialic acid linkage and oligosaccharide backbone sequence. The various types of sialic acid linkage are indicated by the colored panels as defined at the bottom of the figure.

To gain insights into oligosaccharide binding specificities of the five recombinant H5N1 viruses, we performed carbohydrate microarray analyses using a diverse range of sialylated oligosaccharide sequences (Childs et al., 2009; Liu et al., 2010). All five recombinant H5N1 viruses bound to numerous α2-3-sialyl sequences (Fig. 3, Supplementary Table S2). In accord with data from the other two types of SGP-binding assays, the two 134 mutant viruses, rVNCL105(Ala134Val) and rVN1194(Ala134Val), showed markedly lower intensities of binding to the α2-3-sialyl sequences compared with the ‘wild type’ rVN1194 virus, and without any detectable binding to the α2-6-sialyl sequences included in the microarray (Fig. 3). The 186 mutants differed from the ‘wild type’ and the 134 mutants in their ability to bind to α2-6-sialyl sequences. Interestingly, in the microarray analyses, whereby the oligosaccharide probes are presented in a highly clustered state on the solid surface, no reduction was apparent in binding intensities to the α2-3-sialyl sequences by the 186 mutants compared with the ‘wild type’ virus. The reassortant human H3N2 virus, X31, included as an example of virus with HA well-adapted to humans showed, as predicted, preferential binding to α2-6- over α2-3-sialyl sequences.

The four mutant H5N1 viruses resembled the rVN1194 virus in showing high relative binding to the α2-3-sialyl bi-antennary N-glycan (probe ♯61). The multi-antennary α2-3-sialyl N-glycans with or without poly-N-acetyllactosamine extensions were also bound with varying intensities (♯62–♯65). To assist close comparisons of the binding intensities of the five recombinant H5N1 viruses and X31, results are presented in Table 4 for 24 of the sialyl probes that are well matched with respect to backbone sequences, substituents, and aglycones. The 134 mutant viruses showed little or no binding to the α2-3-sialyl sequences, di- and tetra-saccharides of the lacto (type I) or neolacto (type II) series, that lacked fucosylation or sulphation (♯23, ♯25, ♯39, ♯40 and ♯41), whereas the 186 mutants resembled the rVN1194 virus in giving binding signals with these probes. Binding signals were diminished with fucosylation at the penultimate N-acetylglucosamine (GlcNAc) of type I (♯26 vs. ♯23; ♯47 vs. ♯39/LSTa) and type II sequences (♯27 vs. ♯25/3SLN; ♯58 vs. ♯59). On the other hand, probes with sulphation at the penultimate GlcNAc, as on the trisulphated keratan sulfate (♯46) and fucosylated 6-sulpho-sialyl-Lewis x (Le^x^) (♯55), elicited binding with all five H5N1 viruses. Interestingly, the presence of another acidic residue, α2-6-linked sialic acid, at the penultimate GlcNAc also elicited relatively strong binding intensities with ‘wild type’ and mutant viruses (♯107 vs. ♯39). In contrast, sulphation at the terminal galactose, as in the sulphated sialyl-Le^x^ (♯53 and ♯57) elicited binding only with the 186 mutants. The preference of ‘wild type’ and mutant H5N1 viruses for α2-3-sialyl glycans with sulfate or sialic acid on the 6 position of the penultimate GlcNAc residue and their reduced binding to glycans that are fucosylated at this residue is in agreement with findings from previous microarray studies with recombinant HAs of A/Vietnam/1203/2004 (Stevens et al., 2006a) and the H5 consensus sequence (Gambaryan et al., 2004; Wang et al., 2009).

The binding of the two 186 mutants to the biantennary α2-6-sialyl N-glycan (probe ♯98) was less intense than to the α2-3-sialyl analog (♯61), in contrast to X-31. Moreover, the 186 mutants showed little or no binding to many of the α2-6-sialyl sequences bound by X-31, including the trisaccharide probe 6SLN (♯87) and the pentasaccharide probe LSTc (♯90). In contrast to the rVN1194 virus, the 186 mutants, exhibited binding to α2-8-polysialyl sequences (♯122 and ♯123), although this was weaker than that of X31.

In sum, the salient changes observed in the microarray analyses were: first, an overall decrease of α2-3-sialyl binding by the 134 mutants compared with the ‘wild type’ virus while retaining binding to bi- and multiantennary α2-3-sialyl N-glycans and 6-sulphated sequences, but without any detectable α2-6-sialyl binding; and second, the binding of the 186 mutants to the bi-antennary α2-6-sialyl N-glycan but without any apparent reduction in binding to α2-3-sialyl sequences.

### Crystallographic studies

The effects of the single amino acid substitutions in the HA on receptor binding were also studied by X-ray crystallography of HAs in complex with pentasaccharides possessing terminal sialic acid (Sia) linked to galactose (Gal) by either α2-3 (LSTa) or α2-6 (LSTc) linkage.

**Fig. 4:**
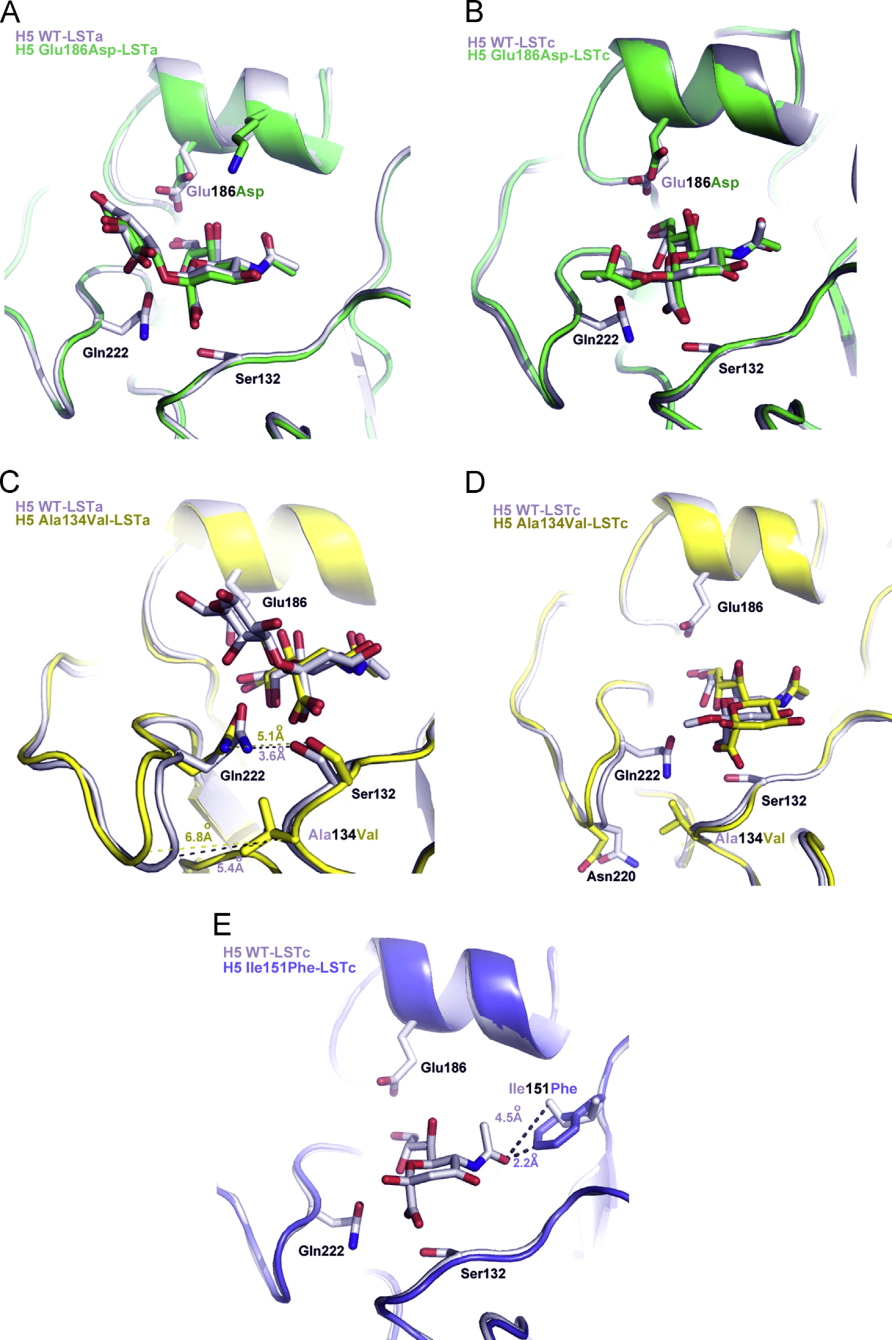
Crystal structures of wild type and mutant H5 hemagglutinins complexed with avian and human receptor analogs (LSTa and LSTc, respectively). (A) Superposition of complexes of LSTa with rVN1194 HA (gray) and with rVN1194(Glu186Asp) HA (green). (B) Superposition of complexes of LSTc with rVN1194 HA (gray) and with rVN1194(Glu186Asp) HA (green). Only density for the Sia-1 of the receptor analog was detected. (C) Superposition of complexes of LSTa with rVN1194 HA (gray) and with rVN1194(Ala134Val) HA (yellow). The increase in width of the hydrophobic channel between the 130 and 220 loops in the mutant that results in an increase in the distance between Gln222 and Ser132 is shown. (D) Superposition of complexes of LSTc with rVN1194 HA (gray) and with rVN1194(Ala134Val) HA (yellow). Only density for the Sia-1 of the receptor analog was detected. E, Superposition of the complex of rVN1194 HA with LSTc (gray) and rVN1194(Ile151Phe) HA (blue). Although no sialic acid was detected in the Ile151Phe crystals soaked in LSTc the proximity of Phe 151 to where the sialic acid would be located is shown.

In the wild type H5 HA complex with the avian receptor analog LSTa, the first three residues, Sia-1, Gal-2, GlcNAc-3 were detected (Ha et al., 2001) but in the Glu186Asp complex only Sia-1 and Gal-2 could be seen (Fig. 4A). This suggests lower affinity of the 186 mutant for the α2-3 sequence. In the wild type HA, Glu186 forms a H-bond with the 9-OH of Sia-1 (Ha et al., 2001). The carboxylate of Asp186 in the mutant is not as close to the 9-OH and as a consequence, the interaction is less tight. The complexes of wild type and Glu186Asp mutant HAs with LSTc showed similar features and indicate only weak binding (Fig. 4B). In both structures, there was clear electron density for only Sia-1, the 2-6-linkage atoms, and two or three additional atoms of Gal-2.

Introduction of a more bulky Val134 side-chain in the mutant, in place of the highly conserved Ala134 in wild type HA, increases the width of the hydrophobic channel between the 130- and 220-loops that form the front and left edges of the receptor-binding site (Fig. 4C). As a consequence, the H-bond between Gln222 and Ser132 is not formed in the mutant. The distances between Gln222 and the glycosidic-linkage O and between Gln222 and the 4-OH of Gal-2 are also slightly increased; as a consequence, the H-bonds formed between Gln222 and LSTa are weaker and Gal-2 was not readily discernible. In the Ala134Val mutant–LSTc complex, only Sia-1 was detected, suggesting slightly weaker binding of the human receptor analog (LSTc), than to the wild type HA (Fig. 4D). These observations are consistent with the comparative binding data obtained with the wild type H5 and the Glu186Asp and Ala134Val mutant recombinant viruses.

Sialic acid was not detected in crystals of the Ile151Phe mutant HA soaked in either LSTa or LSTc receptor analogs. Comparison of the wild type and mutant HAs indicates that the side-chain of Phe151 projects into the receptor binding site about 2 Å further than Ile151 (Fig. 4E). This may restrict receptor binding and consequently impair virus replication.

## Discussion

Changes in the receptor binding characteristics were detected in three of eleven MDCK cell-grown viruses, isolated from upper respiratory tract specimens of influenza H5N1 infected patients, and none of 13H5N1 viruses isolated from poultry. Single amino acid substitutions responsible for the receptor binding changes were identified. Two of the substitutions, Ala134Val and Ile151Phe, were also observed in sequences in the clinical samples from which the isolates were derived, albeit in differing proportions of virus quasispecies compared to MDCK cell isolates, and were therefore likely selected during human infection. In contrast, the Glu186Asp substitution was not detected in the sequence of the clinical specimen and therefore most likely emerged during isolation in MDCK cells, although the presence in the clinical specimen of a minority population below the detection limit of the pyrosequencing method and subsequent selection in cell culture cannot be ruled out.

These observations indicate that H5N1 variants with changing patterns of receptor recognition may rapidly emerge during human H5N1 infections and that further selection of variants in culture systems occurs which may obscure actual evolutionary events during human infection. Precise insights into the effects of observed HA substitutions on receptor recognition are essential to fully understand the evolutionary pathways towards human adaptation of H5N1 viruses, which we addressed using several approaches.

Detailed analyses of the binding properties of the recombinant viruses containing the Ala134Val substitution, observed in virus isolated from a fatal case of H5N1 infection after 4 days of illness, showed consistently that binding to α2-3-sialyl sequences was reduced, but with no detectable increase in binding to the repertoire of α2-6-sialyl sequences examined. The dominant effect of the single 134 substitution was confirmed by the similarity in the binding profiles of the virus with the single substitution and of the virus with the additional 4 substitutions corresponding to the sequence of the MDCK cell isolate. Proportions of 134Val variants were higher in the clinical specimens when compared to the virus isolate (74% vs. 52%), suggesting some reversion to wild-type sequence during culture in α2,3 sialyl-receptor containing MDCK cells. Repeated identification of the Ala134Val substitution in clade 1 human H5N1 isolates (Auewarakul et al., 2007; Li et al., 2004), but not in contemporary clade 1 viruses isolated from birds, emphasizes its potential significance in human infection. A previous report identified the presence of two HA mutations, Ala134Val plus Leu129Val in H5N1 virus from another fatal case in Thailand, which resulted in reduced binding to α2-3-sialyl receptors, but also increased binding to α2-6-sialyl receptors (Auewarakul et al., 2007). Although the Leu129Val substitution alone did not alter the relative binding to the two types of receptor, the effect of the single Ala134Val substitution without the Leu129Val was not described in that report. Tang et al. (2012) recently reported that an Ala134Val substitution in the HA, of a H5N1 isolate from a fatal case in Cambodia, was responsible for reduced binding of HA to MDCK cells and a reduced capacity to agglutinate horse RBCs. In the present instance, no evidence was obtained regarding the possibility that other mutations in HA or other genes within the mixed virus population might complement the properties of the single 134 mutant.

The crystallographic structure of the HA–receptor complexes provided an explanation for the poor receptor binding by the Ala134Val mutant HA, namely that distortion of the binding site by the bulkier valine residue would potentially cause a reduction in the strength of H-bonds between receptor and binding site. The consequent increased width of the site is more akin to that of human H3 (and H9) viruses with their preference for α2-6-sialyl receptors. Whether such changes to the binding site result in improved binding to natural receptors cannot be assessed in the absence of more detailed information as to their identity.

The emergence of the Ile151Phe variant was observed between days 5 and 8 of illness in a patient who ultimately succumbed to infection with an oseltamivir-resistant virus (Tran et al., 2004). The 151Phe variant comprised 65% of the virus population on day 7, increasing to 100% in the clinical specimen from which the MDCK cell isolate was derived on day 8. Unfortunately, recombinant virus containing this substitution could not be propagated in either MDCK cells or eggs, preventing comparable receptor binding analyses. Soaking crystals of the Ile151Phe HA, prepared by vaccinia virus expression, with either the α2-3- or α2-6-sialyl pentasaccharide failed to yield any evidence of ligand binding. The structure of the mutant HA shows that the larger Phe side chain projects further into the binding site such that it may restrict binding. It would appear that this mutation is only advantageous in the presence of compensatory mutations in HA or other genes. In this respect, the possible role of other HA substitutions in this virus relative to VN1194 HA, including Ser133Ala, Leu175Met and Thr188Ala, deserves further research. No other instance of an Ile151Phe substitution in the HA of H5N1 viruses has been reported. However, substitution at this position by threonine (the residue present in human H3 viruses) has been observed in a number of human and avian clade 2.2 viruses isolated between 2004 and 2008, and has become a common feature of a subclade (B1) of Egyptian clade 2.2.1 avian and human viruses since 2009. Although the single Ile151Thr substitution did not affect receptor binding, it was observed to act synergistically with a deletion in residue 129 (also typical of this sublineage) to increase α2-6-sialyl binding (Watanabe et al., 2011).

The Glu186Asp substitution, observed in an MDCK cell-grown virus (but not in the clinical specimen) isolated from a patient who survived the infection, did not markedly alter binding of virus to α2-3-sialyl receptors, in terms of the intensity and spectrum of sequences bound, in the microarray analyses where the oligosaccharide probes are presented in a clustered state, or to immobilized 3SLN-PAA by biolayer interferometry. However, in the third assay procedure with sialoglycopolymer binding to immobilized virus, the Glu186Asp substitution caused a considerable reduction in binding of the low molecular weight trisaccharide 3SLN polymer. This is consistent with poor resolution of the α2-3-sialyl tetrasaccharide, LSTa, in crystals of the HA(Glu186Asp)–LSTa complex. These assay systems using soluble low molecular weight sialoglycopolymer binding to ‘immobilized’ HA thus appear more sensitive in detecting a decrease in affinity for α2-3-sialyl glycans.

A significant change relative to the ‘wild-type’ virus was observed in the binding of the Glu186Asp mutants to α2-6-sialyl sequences in all three types of binding assay. Binding was, however, still less intense than to α2-3-sialyl analogs and relatively poor compared to that of the human H3 virus X31. This was also reflected by the poor resolution of moieties other than the terminal sialic acid in the HA(Glu186Asp)–LSTc complex.

Glu186 is highly conserved, with no substitutions detected among avian or human H5N1 viruses except A/Vietnam/CL1/2004. While the presence of an undetectable minority population cannot be excluded, the absence of 186Asp variants in the clinical specimen as measured by pyrosequencing, strongly suggests that this substitution emerged during MDCK cell culture and illustrates the differences in selective pressures encountered in vitro and in vivo. At any rate, our observations indicate that α2-6-sialyl binding H5N1 variants can emerge during replication in mammalian cells containing these receptors.

The reduction in binding of α2-3-sialyl sequences by the Ala134Val mutant and indication of the same for the Ile151Phe and Glu186Asp mutants is consistent with their loss of ability to agglutinate horse RBCs, which was readily restored on reversion of the mutation on passage (in an avian environment) in eggs. In contrast, there was little discernible effect on agglutination of guinea pig RBCs by the Ala134Val substitution despite weak binding to the α2-6-sialyl sequences included in this study. Although these features suggest specific adaptive evolutionary events in vivo, the significance of the selection of such a phenotype, with reduced binding to avian receptors in the absence of increased preference for human receptors, is not immediately apparent. The microarray encompasses a considerable repertoire of α2-6-sialyl sequences; however, there may be other ‘human-type’ receptor sequences in the respiratory tract that are recognized by the mutant hemagglutinins. Alternatively, the reduced binding to avian receptors may correspond to a phenotype intermediate in adaptation to more human-like characteristics. For example, reduced α2-3-sialyl binding may benefit efficient viral replication in humans by evading the inhibitory effects of α2-3-sialyl glycans present in mucins in the human respiratory tract (Manz et al., 2010). Thus, adaptive evolution of avian influenza H5N1 viruses to efficient binding of human receptors may be a more complex multistep process (Chen et al., 2012). In this respect, it should be noted that the recently reported passage studies in ferrets, which showed that only a limited number of amino acid changes is needed for air-borne transmission of H5N1 viruses, used genetically engineered virus already containing two changes that confer increased α2-6-sialyl binding (Imai et al., 2012; Herfst et al., 2012), changes which did not emerge during passage in ferrets of wild-type viruses. Transmission of the VN1194 mutant was associated with a substantial reduction in affinity for avian α2-3-sialyl receptors and only a slight increase in affinity for human α2-6-sialyl receptors, and Xiong et al. (2013) emphasized the importance of the ratio of preference for the two receptors in determining the biological characteristics. Of note, in addition to the changes in HA that alter receptor specificity, the three human H5N1 viruses investigated (A/Vietnam/CL1/2004, A/Vietnam/CL105/2005, and A/Vietnam/CL2009C/2005) possessed a Glu627Lys change in PB2 (de Jong et al., 2006), the role of which in mammalian adaptation of highly pathogenic avian influenza viruses is well established (Hatta et al., 2001; Le et al., 2009; Shinya et al., 2004; Steel et al., 2009). The detection of multiple adaptive changes in these viruses during human H5N1 infections emphasizes the need for intensive monitoring and vigilance with respect to the continuing threat of H5N1 influenza for global health.

## Material and methods

### Ethics statement

Specimens from patients with H5N1, H1N1 and H3N2 influenza came from existing collections from three observational studies on (a) human H5N1 influenza (de Jong et al., 2005b, 2006); (b) the viral etiology of respiratory tract infections (Do et al., 2011) and (c) on the etiology of viral CNS infections (Le et al., 2010; de Jong et al., 2005a). These studies were approved by the institutional review board of the Hospital for Tropical Diseases, Ho Chi Minh City, and the Oxford Tropical Research Ethical Committee. Informed consent was obtained from all participating patients or their parents or legal guardians.

The poultry specimens also came from existing collections, collected as part of surveillance activities.

### Viruses

Human H5N1 viruses were isolated from nose or throat swabs collected from 11 patients with RT-PCR confirmed H5N1 infection. These patients were admitted to the Hospital for Tropical Diseases in Ho Chi Minh City, Vietnam, during H5N1 outbreaks in early 2004 and 2005 (Tran et al., 2004; Beigel et al., 2005; de Jong et al., 2005b, 2006). In addition, 17 avian influenza viruses (HPAI H5N1, LPAI H4N6 and H6N1) were isolated from tracheal or cloacal swabs collected from poultry (chickens and ducks) in southern Vietnam in the winter of 2006. Ten seasonal human influenza viruses (6H1N1, 4H3N2) served as controls in hemagglutination assays and were isolated from respiratory specimens collected as part of surveillance studies from patients with acute respiratory illness admitted to the Hospital for Tropical Diseases. All swabs were collected in Viral Transport Medium (MEM with Hanks' salts, supplemented with 0.5% gelatin and antibiotics [Sigma-Aldrich]) and stored at −80 °C until further analyses. Initial virus isolation was performed in MDCK cells. Isolated viruses were identified and subtyped by means of hemagglutination-inhibition assays and subtype-specific RT-PCRs as described before (Beigel et al., 2005). All influenza viruses used for the experiments belonged to the same geographical region. H5N1 viruses were all isolated in biosafety level three culture facilities at OUCRU, Vietnam.

### Cells

Madin Darby Canine Kidney (MDCK) cells were cultured in MEM [Eagle's Minimal Essential Medium (Gibco, Invitrogen, USA) containing l-Glutamine and antibiotics]. MDCK-SIAT1 cells, genetically modified to overexpress Neu5Ac2-6 Gal-terminated oligosaccharides, were cultured in MEM containing 1 mg/ml of G-418 antibiotic as previously described (Matrosovich et al., 2003).

### Egg passages

200 μl of virus sample were inoculated into the allantoic cavity of 11-day old embryonated hen eggs. After two days of incubation at 35 °C the allantoic fluid was extracted and virus titrated by hemagglutination assay.

### Preparation of recombinant viruses

Recombinant H5N1 viruses, in which the HA gene of A/Puerto Rico/8/34 (PR8) was replaced by various mutant H5 genes, were generated by reverse genetics as previously described (Hoffmann et al., 2000); the other seven genes were from PR8. A cDNA clone of the HA gene of A/Vietnam/1194/2004 (H5N1), in which the multi-basic cleavage site has been replaced by a single arginine cleavage site (QRERRRKKR to QRETR change), was a gift from Dr. Y. Kawaoka, University of Wisconsin (Yamada et al., 2006). Mutations were introduced into cDNA clones by using a QuikChange site-directed mutagenesis kit (Stratagene, La Jolla, CA) according to the manufacturer's protocol. Two sets of mutant viruses were constructed (Table 3) – one set with single substitutions relative to the HA sequence of A/Vietnam/1194/2004 [rVN1194(Ala134Val) and rVN1194(Glu186Asp)]; the other set with additional substitutions such that the HA sequences correspond (apart from the difference in cleavage site) to the actual sequences of the MDCK cell isolates [rVNCL105(Ala134Val) and rVNCL01(Glu186Asp)]. For rVNCL01(Glu186Asp) an additional substitution was introduced at residue 175 relative to rVN1194(Glu186Asp). For rVNCL105(Ala134Val) and the 151Phe variant the whole HA cDNA was chemically synthesized (GeneArt™ Gene Synthesis, Invitrogen) and cloned into the vector pHW2000 (Hoffmann et al., 2000). Eight plasmids, each containing one of the 8 virus genes, were co-transfected into co-cultured 293T and MDCK cells. After 3–5 days, viruses in the supernatants were recovered by passage in MDCK cells and then grown in the allantoic cavity of 10 day old embryonated hen eggs. The candidate vaccine virus NIBRG14 (Wood and Robertson, 2004), which possesses the HA, with modified cleavage site, (consensus sequence, Table 3) and neuraminidase (NA) from A/Viet Nam/1194/2004 and six internal genes of PR8, was obtained from the National Institute for Biological Standards and Control. The reassortant human H3N2 virus X31 contains the HA and NA of A/Aichi/2/68 and the six internal genes of PR8. Recombinant virus R1 containing the HA and NA of A/Hong Kong/1/68 (H3N2) and remaining genes of A/WSN/33 (H1N1) was generated by reverse genetics (Matrosovich et al., 2007). Viruses were purified by sucrose gradient sedimentation (Skehel and Schild, 1971). The HA genes of all recombinant viruses were sequenced before and after egg growth to confirm their identities.

### Construction of recombinant vaccinia virus and purification of expressed HA

The HA gene encoding the Ile151Phe mutant HA was incorporated into the pRB21 plasmid and recombined with the helper vaccinia virus vRB12 in CV-1 cells to generate a recombinant vaccinia virus encoding the HA, using the system developed by Blasco and Moss (1995). Membrane preparations from recombinant vaccinia virus-infected CV-1 cells were solubilized in 1% dimethyldodecylamine-N-oxide (LDAO) and the HA purified by sucrose gradient centrifugation and ion-exchange chromatography, followed by bromelain digestion, as previously described (Chen et al., 1998).

### Hemagglutination assays

Hemagglutination assays were performed in parallel using two types of red blood cells (RBCs) with differing receptor distributions: horse RBCs which almost exclusively express α2-3-sialyl receptors, and guinea pig RBCs which express both α2-3-sialyl and α2-6-sialyl receptors (Ito et al., 1997a, 1997b; Medeiros et al., 2001). Two-fold dilutions of the virus in phosphate-buffered saline (PBS) at pH 7.2 were mixed in triplicate with equal volumes of guinea pig or horse RBCs diluted in PBS (0.75% and 0.8%, respectively). After 1 h at room temperature the plates were checked for the presence or absence of RBC agglutination and HA titers were determined as the last dilution were detectable hemagglutination was observed.

### RNA extraction and cDNA synthesis

Viral RNA from clinical specimens, cell culture supernatants or allantoic fluids was extracted using a spin-based commercial kit (QIAamp^®^; Qiagen, Germany). The final RNA sample was eluted with 2×50 μl of AVE buffer and stored at −80 °C. The synthesis of complementary DNA from RNA samples was performed using UNI12 primer in a double-step Reverse-Transcription.

### PCR and sequence analysis

A 1027-bp fragment of the HA1 domain of the H5 gene was amplified using the primers: 5′-CGACAGAGCAGGTTGACACAATA-3′ and 5′-GTACCCATACCAACCATCTACCATTC-3′. The generated amplicons were purified using a QIAquick^®^ PCR spin kit (Qiagen, Germany) and were sequenced with the use of a BigDye Terminator Cycle Sequencing Kit version 3.1 (Applied Biosystems), using internal sequence primers. Primers used for sequencing are available upon request. Sequenced fragments were assembled and edited with the use of BioLign version 4.0 (Tom Hall, North Carolina State University). Alignments and residue analyses were performed in BioEdit version 7.0. The sequences of viruses originally isolated in MDCK cells were compared pair wise with those obtained after five passages in the allantois of embryonated hen eggs.

### Pyrosequencing

Biotinylated primers for amplification of HA fragments encompassing mutations of interest (Asp186Glu, Val134Ala and Ile151Phe) and sequencing primers were generated using PSQ assay design software (version 1.0.6, Biotage, Sweden). Primer sequences are available upon request. The HA fragments were amplified in separate PCR reactions in a total volume of 25 μl each, containing 400 mM dNTPs, 1.5 mM MgCL_2_ (Qiagen, Germany), 400 µM of each forward and reverse primer, 1 unit of HotStar Taq (Qiagen, Germany), and 5 µl of cDNA. Biotinylated PCR products were mixed with streptavidin-coated sepharose beads (Amersham Biosciences) followed by treatment with a denaturation solution (Pyro Gold Reagents kit, Biotage) to remove non-biotinylated strands. They were then suspended in annealing buffer (Pyro Gold Reagents kit, Biotage) containing each sequencing primer and subjected to pyrosequencing on a PyroMark™ID instrument according to the manufacturer's instructions (Biotage, Sweden). All resulting pyrograms were automatically analyzed by the PyroMark™ID software version 1.0.5. The limit of detection of viral subpopulations was determined at 10%. To test the depth of coverage, Sanger sequencing was performed on greater than 100 randomly selected bacterial clones. The results revealed a depth of close to 1 in 100.

### Receptor-binding assay using sialylglycopolymers

Synthetic biotinylated sialylglycopolymers (SGPs), 3SLN-PAA and 6SLN-PAA carrying the Neu5Acα2–3Galβ1–4GlcNAc and Neu5Acα2–6Galβ1–4GlcNAc moieties, respectively, were kindly provided by Nicolai Bovin at the Shemyakin and Ovchinnikov Institute of Bioorganic Chemistry, Moscow, Russia. The SGPs contained 20 mol% of the sialyloligosaccharide and 5 mol% of biotin attached to either a 30 kDa or 1 MDa polyacrylamide carrier (Bovin et al., 1993). The binding of SGPs to immobilized viruses using a solid-phase assay was determined as previously described (Matrosovich et al., 2000; Matrosovich and Gambaryan, 2012).

### Biolayer interferometry analysis

Quantitative virus binding experiments were done by biolayer interferometry (BLI) on an Octet RED instrument from Pall ForteBio (Menlo Park, CA, USA), as described (Lin et al., 2012). Briefly, biotinylated 30 kDa sialylglycopolymers 3SLN-PAA and 6SLN-PAA, from Lectinity (Moscow, Russia), were loaded onto streptavidin biosensors (ForteBio) at 0.01–0.5 μg/ml for 5 min in 10 mM HEPES, pH 7.4, 150 mM NaCl, 3 mM EDTA, 0.005% Tween-20. Following a buffer wash, biosensors were incubated with virus solution (100 pM/~2000 HA titer) for 30 min, during which time the association was measured. Experiments were performed at 25 °C and sample plates were agitated at 1000 RPM. Solutions of viruses and all solutions in which biosensors were incubated with virus contained 10 μM oseltamivir carboxylate (Roche) and 10 μM zanamivir (GlaxoSmithKline) to inhibit the viral neuraminidase. Responses at equilibrium were normalized with respect to the maximum response for virus binding and plotted as a function of the amount of sugar immobilized on the biosensor, calculated from the response amplitude during the sugar loading step. Experiments were performed in at least duplicate and the data were pooled for fitting. Relative affinities were estimated from apparent Kds calculated from the fractional occupancy.

### Carbohydrate microarray analyses

Purified viruses were inactivated by treatment with β-propiolactone (0.166% v/v) for 40 min at 33 °C. Viruses were pelleted by centrifugation at 100,000*g* for 30 min and resuspended in 0.01 M phosphate buffered saline pH 7.4 (PBS) containing 0.05% sodium azide. The β-propiolactone has been shown previously not to affect the binding profile of viruses in microarray analyses (Childs et al., 2009), and did not affect the hemagglutination titers of the H5N1 viruses using turkey, guinea pig or horse erythrocytes (not shown).

The virus concentrations were adjusted so that the stock preparations had equal HA glycoprotein content. This was determined for the various purified virus preparations by densitometric measurement of the HA band in Coomassie blue stained SDS polyacrylamide gels run under non-reducing conditions. A virus standard, where the total viral protein content had been determined using a BCA protein assay kit (Thermo Fisher Scientific, Cramlington, UK), was included on the gel and virus concentrations are expressed as mg total virus protein per ml.

Carbohydrate microarray analyses were performed, essentially as described (Childs et al., 2009), using a new version of oligosaccharide microarrays (Glycosciences Array Set 40-41) containing six neutral and 119 sialylated, lipid-linked oligosaccharide probes (Supplementary Table S2). In brief, the viruses were suspended in 20 mM HEPES buffer pH 7.2 containing 150 mM NaCl, 5 mM CaCl_2_, 0.15 µM zanamivir (a gift from GlaxoSmithKline) (HBS-Ca^2+^/Z), and 0.2% (w/v) bovine serum albumin (BSA, fatty acid and globulin free, Sigma A0281). The solution for blocking non-specific binding and as a diluent for reagents in the analyses was HBS-Ca^2+^/Z containing 2% (w/v) BSA. In the present investigations H5N1 viruses were analyzed at 1 mg HA protein/ml and X31 virus at 0.6 mg HA protein/ml. Binding of the H5N1 viruses was detected with a hyperimmune ferret anti-NIBRG14 serum, and of X31 with a rabbit antiserum to this virus, at 1:150 dilution, followed by biotin-conjugated protein A (10 µg/ml) and Alexa Fluor 647-conjugated Streptavidin A (1 µg/ml). In the absence of viruses, no significant signals were given by the antisera. The results presented in Fig. 3 are representative of at least two independent analyses with each virus.

### Crystallographic methods

Wild type and mutant HAs were prepared by bromelain digestion of purified viruses or HA purified from membrane preparations of vaccinia virus-infected cells, as previously described (Chen et al., 1998; Ha et al., 2002). Crystallization conditions were as reported for NIBRG14 BHA (Yamada et al., 2006) and crystals with bound receptor analogs were prepared by soaking the BHA crystals overnight in either 4 mM LSTa (NeuAcα2–3Galβ1–3GlcNAcβ1–3Galβ1–4Glc) or 4 mM LSTc (NeuAcα2–6Galβ1–4GlcNAcβ1–3Galβ1–4Glc) in cryo buffer. All of the data were collected at the Diamond Light Source at 100 K and were integrated using Denzo and scaled with Scalepack. Standard refinement, with Refmac and PHENIX, and manual model building with Coot was performed on all of the structures. The refinement statistics and the relative real space correlation coefficients of the ligand are given in Supplementary Tables S3, S4, S5, S6 and S7. The figures (panels in Fig. 4) were created with Pymol.

## Additional information

### Accession codes

Coordinates and structure factors were deposited in the PDB database under accession codes: 3ZP0 (rVN1194 H5 HA with LSTa), 3ZP1 (rVN1194 H5 HA with LSTc), 3ZP2 (rVN1194(Ala138Val) H5 HA mutant with LSTa), 3ZP3 (rVN1194(Ala138Val) H5 HA mutant with LSTc), 3ZPb (rVN1194(Glu190Asp) H5 HA mutant with LSTa), 3ZP6 (rVN1194(Glu190Asp) H5 HA mutant with LSTc), and 3ZPa (rVN1194(Ile155Phe) H5 HA mutant). Note: The amino acid residue numbering of the deposited structures is based on the H3 sequence (e.g. 138Val, 190Asp and 155Phe) and not on the H5 sequence (e.g. 134Val, 186Asp and 151Phe) selected here.

## Figures and Tables

**Table 1:** Hemagglutination titers of MDCK cell- and egg-cultured Influenza A viruses.

**Virus**	**Subtype**	**Origin**	**MDCK GMT**^a^		**ω5 GMT**^a^	
			**Horse**	**Guinea pig**	**Horse**	**Guinea pig**
A/VN/HTD33/04	H1N1	Human	0	75	0	24
A/VN/HTD34/04	H1N1	Human	0	192	0	32
A/VN/HTD566/04	H3N2	Human	0	53	0	4
A/VN/HTD567/04	H3N2	Human	0	21	0	8
A/Ck/VLC13/06	H5N1	Chicken	267	144	12	32
A/Ck/VLC15/06	H5N1	Chicken	491	416	64	24
A/Dk/LAD404/06	H4N6	Duck	341	352	48	128
A/Dk/DTD516/06	H6N1	Duck	224	160	32	32
A/VN/CL1/04	H5N1	Human	0	100	512	192
A/VN/CL2/04	H5N1	Human	52	72	64	48
A/VNCL26/04	H5N1	Human	64	192	256	192
A/VN/CL36/04	H5N1	Human	64	68	64	64
A/VN/PEV016/04	H5N1	Human	32	44	128	96
A/VN/CL100/05	H5N1	Human	112	352	192	384

A/VN/CL105/05	H5N1	Human	0	120	128	320
A/VN/CL107/05	H5N1	Human	112	464	NA^b^	NA^b^
A/VN/CL115/05	H5N1	Human	112	200	8	20
A/VN/CL119/05	H5N1	Human	80	136	384	384
A/VN/CL2009C/05	H5N1	Human	27	405	128	96

ω5: five serial passages in the allantois of embryonated hen eggs.

a Geometric mean titers of triplicates.

b Not analyzed due to technical difficulties.

**Table 2:** Amino acid residues in MDCK cell- and egg-cultured viruses and viruses in clinical specimens.

**Virus isolates and clinical samples (day since onset of illness)**	**HA position**	**Amino acid (%)**^a^	
**Wild-type**^b^	**Mutant**
A/VN/CL1/04	186	Glu (5)	Asp (95)
A/VN/CL1/04 ω5	186	Glu (97)	Asp (3)
CL1 Throat swab (d8)^c^	186	Glu (100)	Asp (0)
CL1 Nose swab (d10)	186	Glu (100)	Asp (0)

A/VN/CL2009C/05	151	Ile (3)	Phe (95), Leu (2)
A/VN/CL2009C/05 ω5	151	Ile (0)	Phe (2), Leu (98)
CL2009 Throat swab (d7)	151	Ile (35)	Phe (65)
CL2009 Nose swab (d8)	151	Ile (0)	Phe (100)
CL2009 Throat swab (d8)^c^	151	Ile (0)	Phe (100)

A/VN/CL105/05	134	Ala (48)	Val (52)
A/VN/CL105/05 ω5	134	Ala (96)	Val (4)
CL105 Throat swab (d4)^c^	134	Ala (26)	Val (74)
CL105 Tracheal aspirate (d4)	134	Ala (27)	Val (73)

ω5: five serial passages in the allantois of embryonated chicken eggs.

a Proportions of subpopulations were determined by pyrosequencing of the regions of interest.

b Wild-type refers to the avian virus consensus sequence.

c Source of the MDCK cell isolate.

**Table 3:** Amino acid differences and hemagglutination patterns with guinea pig and horse red blood cells of recombinant H5N1 viruses^a^.

**Viruses**	**HA residue number**						**Hemagglutination titer**^b^	
83	94	134	175	186	188	Guinea pig	Horse
NIBRG14 (rVN1194)	Ala	Asp	**Ala**	Leu	**Glu**	Thr	256	128

rVNCL105 (Ala134Val)	Val	Val	**Val**	Ile		Ile	256	4
rVN1194 (Ala134Val)			**Val**				1024	<4
rVNCL01 (Glu186Asp)				Met	**Asp**		1024	<4
rVN1194 (Glu186Asp)					**Asp**		512	<4

a The residue designation is according to H5 numbering. Differences from the sequence of VN1194 are shown. Amino acids in positions that were altered on egg passage are highlighted in bold.

b Using the virus-containing allantoic fluid before purification of the viruses for the microarray analyses.

**Table 4:** Virus binding to 24 oligosaccharide probes that are well matched with respect to backbone sequences, substituents and aglycones in the carbohydrate microarrays.

## Footnotes

Appendix A Supplementary data associated with this article can be found in the online version at doi:10.1016/j.virol.2013.08.010.
