# Supplementary material for: Changes in the hemagglutinin of H5N1 viruses during human infection – Influence on receptor binding
Source: Virology. 2013 Dec;447(1-2):326–37. doi: 10.1016/j.virol.2013.08.010 (PMC3820038; doi:10.1016/j.virol.2013.08.010)
Supplement: Supplementary file 1 — Supplementary Data [file mmc1.pdf]

Changes in the Hemagglutinin of H5N1 Viruses during Human Infection – Influence on Receptor Binding

Martin Crusat, Junfeng Liu, Angelina S. Palma, Robert A. Childs, Yan Liu, et al

SUPPLEMENTARY INFORMATION

Table S1 Hemagglutination titers of additional MDCK cell-cultured human and avian Influenza A viruses.

Table S2 Oligosaccharide probes[a] included in the microarrays, sorted by sialyl linkage and backbone sequences, and the binding signals (fluorescence intensities) they elicited with the six viruses, rVN1194, rVNCL105(Ala134Val), rVN1194(Ala134Val), rVNCL01(Glu186Asp), rVN1194(Glu186Asp) and X31.

Table S3 The relative real space correlation coefficient of the ligand to Trp149.

Table S4 Data collection and refinement statistics of rVN1194 H5 HA.

Table S5 Data collection and refinement statistics of rVN1194(Ala134Val) H5 HA mutant.

Table S6 Data collection and refinement statistics of rVN1194(Glu186Asp) H5 HA mutant.

Table S7 Data collection and refinement statistics of rVN1194(Ile151Phe) H5 HA mutant

Table S1 Hemagglutination titers of additional MDCK cell-cultured human and avian Influenza A viruses.

Virus	Subtype	Origin	Horse	Guinea pig
A/VN/HTD538/04	H1N1	Human	0	192
A/VN/HTD541/04	H1N1	Human	0	213
A/VN/HTD547/04	H1N1	Human	0	128
A/VN/CHY/04	H1N1	Human	0	128
A/VN/HTD548/04	H3N2	Human	0	27
A/VN/HTD558/04	H3N2	Human	0	32
A/Ck/VLC1/06	H5N1	Chicken	277	176
A/Ck/VLC2/06	H5N1	Chicken	170	120
A/Ck/VLC3/06	H5N1	Chicken	160	152
A/Ck/VLC4/06	H5N1	Chicken	203	140
A/Ck/VLC11/06	H5N1	Chicken	427	216
A/Ck/VLC12/06	H5N1	Chicken	149	60
A/Ck/VLC16/06	H5N1	Chicken	235	240
A/Ck/VLC19/06	H5N1	Chicken	139	76
A/Ck/VLC21/06	H5N1	Chicken	139	144
A/Ck/VLC22/06	H5N1	Chicken	821	184
A/Ck/VLC24/06	H5N1	Chicken	501	184

[†]Geometric mean titers of triplicates

Table S2 Oligosaccharide probes[a] included in the microarrays, sorted by sialyl linkage and backbone sequences, and the binding signals (fluorescence intensities) they elicited with the six viruses, rVN1194, rVNCL105(Ala134Val), rVN1194(Ala134Val), rVNCL01(Glu186Asp), rVN1194(Glu186Asp) and X31.

ID	*Sequence*	Name	Fluorescence signal intensities[b] at 5 fmol per spot					
			rVN1194	rVNCL105 Ala134Val	rVN1194 Ala134Val	rVNCL01 Glu186Asp	rVN1194 Glu186Asp	X31
	Neutral							
1	Galß-4Glc-AO	Lac-AO	-	-	-	1309	91	-
2	Galß-4GlcNAc-AO	LacNAc-AO	-	-	34	330	94	-
3	Galß-3GlcNAcß-3Galß-4Glc-DH	LNT	35	-	-	-	36	14
4	Galß-4GlcNAcß-3Galß-4Glc-DH	LNnT	17	23	7	-	30	-
5	Galß-4GlcNAcß-3Galß-4Glc-DH<br>| Fucα-3	LNFP-III	31	10	-	73	60	13
6	Galß-4GlcNAcß-2Manα-6<br>| Manß-4GlcNAcß-4GlcNAc-DH<br>| Galß-4GlcNAcß-2Manα-3	NA2	24	13	15	93	3	-
	2-3 Sialyl<br>***Gal, Lactose and N-acetyllactosamine-based linear backbones***							
7	NeuAcα-3Galβ-Cer	GM4	92	269	27	350	-	36
8	NeuAcα-3Galß-4Glcß-Cer	GM3	91	-	23	-	148	60
9	NeuGcα-3Galß-4Glcß-Cer	GM3(Gc)	-	2	-	-	42	-
10	NeuAcα-3Galß-4Glcß-Cer	Haematoside	6020	-	-	978	360	-
11	NeuAcα-3Galß-4Glc-AO	NeuAcα-(3')Lac-AO	683	55	-	1783	3587	-
12	KDNα-3Galβ-4Glcß-C30	GSC-199	-	-	-	-	-	16
13	NeuAcß-3Galß-4Glc-AO	NeuAcß-(3')Lac-AO	-	-	-	-	79	27
14	Neuα-3Galß-4Glc-AO	Neuα-(3')Lac-AO	-	-	-	-	-	-
15	Neu4,5Acα-3Galß-4Glc-AO	Neu4,5Ac-(3')Lac-AO	703	250	28	5533	10174	12
16	(4-deoxy)NeuAcα-3Galß-4Glcß-Cer36	GSC-75	13	-	107	449	55	5
17	(7-deoxy)NeuAcα-3Galß-4Glcß-Cer36	GSC-76	167	143	127	434	208	121
18	(8-deoxy)NeuAcα-3Galß-4Glcß-Cer36	GSC-77	10	9	-	129	190	-
19	(9-deoxy)NeuAcα-3Galß-4Glcß-Cer36	GSC-51	39	2	70	323	65	5
20	(4-OMe)NeuAcα-3Galß-4Glcß-Cer36	GSC-78	24	2	-	714	145	-
21	(9-OMe)NeuAcα-3Galß-4Glcß-Cer36	GSC-79	41	11	-	183	70	-
22	NeuAcα-3Galß-4Glcß-C30<br>| Fucα-3	GSC-161	10	45	-	1567	714	-
23	NeuAcα-3Galß-3GlcNAc-AO	NeuAcα-(3')LN1-3-AO	3841	321	40	8869	10706	228
24	NeuAcα-3Galß-4GlcNAc-DH	NeuAcα-(3')LN	-	-	-	-	-	35

25	NeuAcα-3Galß-4GlcNAc-AO	NeuAcα-(3')LN-AO	872	55	-	3198	4044	67
26	NeuAcα-3Galß-3GlcNAc-AO<br>Fucα-4	SA(3')-Lea-Tri-AO	759	64	26	4363	6874	44
27	NeuAcα-3Galß-4GlcNAc-AO<br>Fucα-3	SA(3')-Lex-Tri-AO	71	-	-	502	924	62
28	NeuAcα-3Galß-4GlcNAcß-C30<br>Fucα-3	GSC-440	-	1	-	171	31	19
29	Neu4,5Acα-3Galß-4GlcNAcß-C30<br>Fucα-3	GSC-512	55	11	-	223	81	-
30	Neu5,9Acα-3Galß-3GlcNAcß-C30<br>Fucα-4	GSC-513	10	-	-	310	-	-
31	Neu5,9Acα-3Galß-4GlcNAcß-C30<br>Fucα-3	GSC-511	-	658	-	283	234	-
	2-3 Sialyl<br>Lacto-N-neotetraose and Lacto-N-tetraose-based linear backbones							
32	NeuAcα-3Galß-4GlcNAcß-3Galß-C30<br>Fucα-3	GSC-479	257	91	22	3302	2646	64
33	NeuAcα-3Galß-4GlcNAcß-3Galß-Cer36<br>Fucα-3	GSC-105	186	43	6	768	142	28
34	NeuGcα-3Galβ-4GlcNAcβ-3Galß-Cer36<br>Fucα-3	GSC-177	-	-	50	199	63	24
35	KDNα-3Galβ-4GlcNAcβ-3Galß-C30<br>Fucα-3	GSC-341	-	-	-	160	39	-
36	NeuAcα-3(4,6-deoxy)Galß-4GlcNAcß-3Galß-Cer36<br>Fucα-3	GSC-257	47	-	-	266	79	276
37	NeuAcα-3(4-deoxy)Galß-4GlcNAcß-3Galß-Cer36<br>Fucα-3	GSC-175	46	15	-	340	32	372
38	NeuAcα-3(6-deoxy)Galß-4GlcNAcß-3Galß-Cer36<br>Fucα-3	GSC-176	116	34	-	742	80	26
39	NeuAcα-3Galß-3GlcNAcß-3Galß-4Glc-DH	LSTa	4884	598	452	11255	17761	913
40	NeuAcα-3Galβ-3GlcNAcβ-3Galβ-4Glcß-C30	GSC-272	11368	513	238	36466	33320	6289
41	NeuAcα-3Galβ-4GlcNAcβ-3Galβ-4Glcß-C30	GSC-273	8016	379	198	31552	24121	3009
42	NeuGcα-3Galβ-3GlcNAcβ-3Galβ-4Glcß-C30	GSC-396	-	-	60	368	101	-
43	NeuAcα-3Galß-4GlcNAcß-3Galß-4Glcß-Cer	Sialylparagloboside	3093	181	45	2314	4147	166
44	NeuAcα-3Galß-4GlcNAcß-3Galß-4Glcß-Cer36	GSC-31	265	34	-	1487	1099	2
45	Neuα-3Galß-4GlcNAcß-3Galß-4Glcß-Cer36<br>SU-6	GSC-516B	30	3	-	416	38	34

46	NeuAcα-3Galβ-4GlcNAcβ-3Galβ-3GlcNAc-DH<br>SU-6 (on Galβ), SU-6 (on GlcNAcβ), SU-6 (on Galβ)	C4U	25180	3086	801	33304	49063	2308
47	NeuAcα-3Galß-3GlcNAcß-3Galß-4Glc-DH<br>Fucα-4 (on GlcNAcß)	SA(3')-LNFP-II	473	195	134	2352	4738	291
48	NeuAcα-3Galß-4GlcNAcß-3Galß-4Glc-DH<br>Fucα-3 (on GlcNAcß)	SA(3')-LNFP-III	1677	1062	1045	10083	14261	886
49	NeuAcα-3Galß-4GlcNAcß-3Galß-4Glcß-Cer36<br>Fucα-3 (on GlcNAcß)	GSC-64	284	107	-	798	1840	123
50	NeuAcα-3Galß-4GlcNß-3Galß-4Glcß-Cer36<br>Fucα-3 (on GlcNß)	GSC-533	558	133	-	2076	1351	43
51	KDNα-3Galβ-4GlcNAcβ-3Galβ-4Glcß-Cer36<br>Fucα-3 (on GlcNAcβ)	GSC-149	-	4	34	456	85	12
52	Neuα-3Galß-4GlcNAcß-3Galß-4Glcß-Cer36<br>Fucα-3 (on GlcNAcß)	GSC-472	-	20	30	203	-	17
53	SU-6 (on Galß)<br>NeuAcα-3Galß-4GlcNAcß-3Galß-4Glcß-Cer36<br>Fucα-3 (on GlcNAcß)	GSC-268	66	101	50	3047	8926	151
54	SU-6 (on Galß)<br>Neuα-3Galß-4GlcNß-3Galß-4Glcß-Cer36<br>Fucα-3 (on GlcNß)	GSC-268 deNAc	19	8	5	30	-	-
55	SU-6 (on GlcNAcß)<br>NeuAcα-3Galß-4GlcNAcß-3Galß-4Glcß-Cer36<br>Fucα-3 (on GlcNAcß)	GSC-269	2280	1130	1064	8163	10462	450
56	SU-6 (on GlcNAcß)<br>Neuα-3Galß-4GlcNAcß-3Galß-4Glcß-Cer36<br>Fucα-3 (on GlcNAcß)	GSC-406	-	13	-	-	72	37
57	SU-6 (on Galß), SU-6 (on GlcNAcß)<br>NeuAcα-3Galß-4GlcNAcß-3Galß-4Glcß-Cer36<br>Fucα-3 (on GlcNAcß)	GSC-270	357	255	106	6150	15234	182
58	NeuAcα-3Galß-4GlcNAcß-3Galß-4GlcNAcß-3Galß-4Glcß-Cer36<br>Fucα-3 (on first GlcNAcß), Fucα-3 (on second GlcNAcß)	GSC-220	2146	357	-	2421	6399	507
59	NeuAcα-3Galß-4GlcNAcß-3Galß-4GlcNAcß-3Galß-4Glcß-Cer36<br>Fucα-3 (on second GlcNAcß)	GSC-221	4576	407	123	4325	11085	1547
	2-3 Sialyl<br>***Branched backbone***							

60	Galß-4GlcNAcß-6<br>Fucα-3 Galß-4Glc-DH<br>NeuAcα-3Galß-3GlcNAcß-3	MSMFLNH	9152	832	1264	18421	21915	3167
	2-3 Sialyl N-glycans							
61	NeuAcα-3Galß-4GlcNAcß-2Manα-6 Fucα-6<br>Manß-4GlcNAcß-4GlcNAc-DH<br>NeuAcα-3Galß-4GlcNAcß-2Manα-3	A2F(2-3)	30469	4042	4939	36210	49408	14413
62	NeuAcα-3Galß-4GlcNAcß-6<br>NeuAcα-3Galß-4GlcNAcß-2Manα-6 Fucα-6<br>Manß-4GlcNAcß-4GlcNAc-DH<br>NeuAcα-3Galß-4GlcNAcß-2Manα-3	P22-1	20776	1054	3537	31785	33393	3928
63	NeuAcα-3Galß-4GlcNAcß-6<br>NeuAcα-3Galß-4GlcNAcß-2Manα-6 Fucα-6<br>Manß-4GlcNAcß-4GlcNAc-DH<br>NeuAcα-3Galß-4GlcNAcß-2Manα-3<br>NeuAcα-3Galß-4GlcNAcß-4	P6-1	15379	591	1322	16326	26582	662
64	NeuAcα-3Galß-4GlcNAcß-6<br>NeuAcα-3Galß-4GlcNAcß-3Galß-4GlcNAcß-2Manα-6 Fucα-6<br>Manß-4GlcNAcß-4GlcNAc-DH<br>NeuAcα-3Galß-4GlcNAcß-2Manα-3<br>NeuAcα-3Galß-4GlcNAcß-4	P7-2	18621	807	1556	19013	25773	897
65	NeuAcα-3Galß-4GlcNAcß-3Galß--4GlcNAcß-6<br>NeuAcα-3Galß-4GlcNAcß-3Galß-4GlcNAcß-2Manα-6 Fucα-6<br>Manß-4GlcNAcß-4GlcNAc-DH<br>NeuAcα-3Galß-4GlcNAcß-2Manα-3<br>NeuAcα-3Galß-4GlcNAcß-4	P8-1	44179	3553	845	38697	43250	2958
	2-3 Sialyl Ganglioside related							
66	GalNAcβ-4Galβ-4Glcβ-Cer<br>NeuAcα-3	GM2	-	40	-	-	16	28

67	Galβ-3GalNAcβ-4Galβ-4Glcβ-Cer<br>| NeuAcα-3	GM1	9	-	39	686	-	-
68	Galβ-3GalNAcβ-4Galβ-4Glc-DH<br>| NeuAcα-3	GM1-penta	-	509	-	290	-	-
69	Galβ-3GalNAcβ-4Galβ-4Glcβ-Cer<br>| NeuGcα-3	GM1(Gc)	-	30	-	121	-	-
70	Galβ-3GalNAcβ-4Galβ-4Glc-DH<br>| NeuGcα-3	GM1(Gc)-penta	73	-	-	384	55	11
71	KDNα-3Galβ-3GalNAcβ-4Galβ-4Glcß-Cer36<br>| KDNα-3	GSC-195	-	-	28	232	101	-
72	NeuAcα-3Galß-3GalNAcß-4Galß-4Glcß-Cer<br>| NeuAcα-3	GD1a	779	57	-	684	2181	47
73	NeuAcα-3Galß-3GalNAcß-4Galß-4Glc-DH<br>| NeuAcα-3	GD1a-hexa	3105	133	23	4312	7318	118
74	SU-6<br>| NeuAcα-3Galß-3GalNAcß-4Galß-4Glcß-Cer36	GSC-335	890	12	-	2064	1053	203
	2-3 Sialyl<br>***O-glycan related***							
75	NeuAcα-3Galß-3GalNAcß-C30	GSC-488	-	-	-	82	36	-
76	SU-6<br>| NeuAcα-3Galß-3GalNAcß-C30	GSC-489	53	-	-	112	24	-
77	NeuAcα-3Galß-4GlcNAcß-6Galß-4Glcß-Cer36<br>| Fucα-3	GSC-154	-	16	7	6	54	-
	2-3 Sialyl<br>***Miscellaneous***							
78	NeuAcα-3Galß-4GlcNAcß-6GalNAcα-3Galß-4Glcß-C30	GSC-441	762	105	21	5839	7597	296
79	NeuAcα-3Galß-4GlcNAcß-4GalNAcß-3Galß-4Glcß-C30<br>| Fucα-3	GSC-384	174	81	12	4209	4219	479
80	GalNAcβ-6Galβ-4Glcß-Cer36<br>| NeuAcα-3	GSC-284	-	11	-	420	199	-
	2-6 Sialyl<br>***Gal, Lactose and N-acetyllactosamine-based linear backbones***							
81	NeuAcα-6Galß-Cer36	GSC-27	7	4	16	55	12	-
82	NeuAcα-6Galß-4Glcß-Cer36	GSC-61	-	8	40	35	-	11
83	NeuAcα-6Galß-4Glc-AO	NeuAcα-(6')Lac-AO	-	-	7	942	59	1190
84	NeuAcß-6Galß-4Glc-AO	NeuAcß-(6')Lac-AO	-	-	-	-	-	-
85	Neuα-6Galß-4Glc-AO	Neuα-(6')Lac-AO	206	-	-	1789	39	-
86	NeuAcα-6Galß-4GlcNAc	NeuAcα-(6')LN	16	-	-	-	50	139

87	NeuAcα-6Galß-4GlcNAc-AO	NeuAcα-(6')LN-AO	-	-	14	29	9	4100
88	Neu5,9Acα-6Galß-4GlcNAc-DH	Neu5,9Ac-(6')LN	-	-	17	590	-	-
	2-6 Sialyl<br>***Lacto-N-neotetraose and Lacto-N-tetraose-based linear backbones***							
89	Galβ-3GlcNAcβ-3Galβ-4Glc-DH<br>| NeuAcα-6	LSTb	44	-	-	-	-	-
90	NeuAcα-6Galβ4-GlcNAcβ3-Galβ4-Glc-DH	LSTc	-	29	-	436	764	18831
91	NeuGcα-6Galβ-3GlcNAcβ-3Galβ-4Glcß-C30	GSC-397	-	-	16	107	-	792
92	NeuAcα-6Galß-4GlcNAcß-3Galß-4Glcß-Cer36<br>| Fucα-3	GSC-97	25	17	84	70	1006	1726
93	NeuAcα-6Galß-4GlcNAcß-3Galß-4Glc-DH<br>| Fucα-3	SA(6')-LNFP-VI	13	9	-	163	387	13407
	2-6 Sialyl<br>***Branched backbone***							
94	NeuAcα-6Galß-4GlcNAcß-6<br>| Galß-4Glc-DH<br>| Galß-3GlcNAcß-3	MSLNH	4	11	38	64	72	5729
95	Galß-4GlcNAcß-6<br>| Galß-4Glc-DH<br>| NeuAcα-6Galß-3GlcNAcß-3	MSLNnH-I	-	25	66	915	1686	14983
96	NeuAcα-6Galß-4GlcNAcß-6<br>| Galß-4Glc-DH<br>| NeuAcα-6Galß-4GlcNAcß-3	DSLNnH	7	2	57	1045	4530	11709
97	Galß-4GlcNAcß-6<br>| | Fucα-3 Galß-4Glc-DH<br>| NeuAcα-6Galß-3GlcNAcß-3	MFMSLNnH	-	73	-	272	1199	20367
	2-6 Sialyl<br>***N-glycans***							
98	NeuAcα-6Galß-4GlcNAcß-2Manα-6<br>| Manß-4GlcNAcß-4GlcNAc-DH<br>| NeuAcα-6Galß-4GlcNAcß-2Manα-3	A2(2-6)	156	14	7	8768	18025	30875
	2-6 Sialyl<br>***Ganglioside related***							
99	GalNAcβ-4Galβ-4Glcß-Cer36<br>| NeuAcα-6	GSC-442	55	10	-	49	-	-
100	NeuAcα-6Galß-3GalNAcß-4Galß-4Glcß-Cer36	GSC-68	-	-	48	222	61	11934

101	Galβ-3GalNAcβ-4Galβ-4Glcß-Cer36<br>NeuAcα-6	GSC-155	-	16	-	217	-	23
102	NeuAcα-6Galβ-3GalNAcβ-4Galβ-4Glcß-Cer36<br>NeuAcα-6	GSC-107	-	5	12	288	36	2047
	2-6 Sialyl<br>Miscellaneous							
103	NeuAcα-6Galß-6GalNAcß-4Galß-4Glcß-Cer36	GSC-70	8	31	41	164	-	486
	2-3 and 2-6 Sialyl<br>O-glycan related							
104	NeuAcα-3Galß-3GalNAc-DH<br>NeuAcα-6	DST	214	19	-	2110	4227	62
105	NeuAcα-3Galß-3GalNAc-AO<br>NeuAcα-6	DST-AO	1739	39	-	3346	3665	-
106	NeuAcα-3Galß-3GalNAcß-C30<br>NeuAcα-6	GSC-490	-	4	17	27	24	-
	2-3 and 2-6 Sialyl<br>Lacto-N-neotetraose and Lacto-N-tetraose-based linear backbone							
107	NeuAcα-3Galß-3GlcNAcß-3Galß-4Glc-DH<br>NeuAcα-6	DSLNT	14208	1409	378	16686	34257	1132
	2-3 and 2-6 Sialyl<br>N-glycan							
108	NeuAcα-3Galß-4GlcNAcß-2Manα-6<br>Manß-4GlcNAcß-4GlcNAc-DH<br>NeuAcα-3Galß-4GlcNAcß-4Manα-3<br>NeuAcα-6Galß-4GlcNAcß-2	A3	22147	2083	358	29544	36228	19599
	2-3 and 2-6 Sialyl<br>Ganglioside related							
109	NeuAcα-3Galβ-3GalNAcβ-4Galβ-4Glcß-Cer36<br>NeuAcα-6	GSC-118	322	103	15	670	311	14
	2-8 Sialyl<br>Gal, Lactose and N-acetyllactosamine-based linear backbones							
110	NeuAcα-8NeuAcα-3Galß-Cer36	GSC-230	22	-	-	20	11	13
111	NeuAcα-8NeuAcα-6Galß-Cer36	GSC-231	-	-	-	194	-	24
112	NeuAcα-8NeuAcα-8NeuAcα-6Galß-Cer36	GSC-439	-	7	-	132	-	42
	2-8 Sialyl<br>Ganglioside related							
113	NeuAcα-8NeuAcα-3Galß-4Glcß-Cer	GD3	30	-	-	1103	141	-
114	NeuAcα-8NeuAcα-3Galß-4Glc-AO	GD3-tetra-AO	-	1	-	6	234	1892

115	NeuAcα-8NeuAcα-3Galß-4Glcß-Cer36	GSC-229	26	-	-	144	1	169
116	NeuAcα-8NeuAcα-8NeuAcα-3Galβ-4Glcß-Cer36	GSC-437	-	11	-	48	5	264
117	GalNAcß-4Galß-4Glcß-Cer<br>| NeuAcα-8NeuAcα-3	GD2	-	35	-	-	65	-
118	Galß-3GalNAcß-4Galß-4Glcß-Cer<br>| NeuAcα-8NeuAcα-3	GD1b	-	-	-	105	-	-
119	NeuAcα-8NeuAcα-3Galβ-3GalNAcβ-4Galβ-4Glcβ-Cer<br>| NeuAcα-8NeuAcα-3	GQ1b	172	123	-	-	205	2393
	2-8 Sialyl<br>***Polysialyl***							
120	NeuAcα-8NeuAcα-8NeuAc-DH	SA3(α8)	-	-	-	82	-	548
121	NeuAcα-8NeuAcα-8NeuAcα-8NeuAcα-8NeuAc-DH[c]	SA5(α8)	-	-	98	529	263	12527
122	NeuAcα-8NeuAcα-8NeuAcα-8NeuAcα-8NeuAcα-8NeuAcα-8NeuAc-DH[c]	SA7(α8)	-	23	-	2077	1743	18260
123	NeuAcα-8NeuAcα-8NeuAcα-8NeuAcα-8NeuAcα-8NeuAc-8NeuAcα-8NeuAc-<br>8NeuAcα-DH[c]	SA9(α8)	-	41	-	2093	2538	19071
	2-3 and 2-8 Sialyl<br>***Ganglioside related***							
124	NeuAcα-8NeuAcα-3Galβ-3GalNAcβ-4Galβ-4Glcβ-Cer<br>| NeuAcα-3	GT1a	61	-	-	720	117	3703
125	NeuAcα-3Galβ-3GalNAcβ-4Galβ-4Glcβ-Cer<br>| NeuAcα-8NeuAcα-3	GT1b	4745	125	88	941	4520	80
	2-9 Sialyl<br>***Gal, Lactose and N-acetyllactosamine-based linear backbones***							
126	NeuAcα-9NeuAcα-3Galβ-4Glcß-Cer36	GSC-96	-	-	35	163	57	14

[a] The oligosaccharide probes are all lipid-linked, and are from the collection assembled in the course of research in Glycosciences Laboratory. DH, designates NGLs prepared from reducing oligosaccharides by reductive amination with the amino lipid, 1,2-dihexadecyl-*sn*-glycero-3-phosphoethanolamine (DHPE) (1); AO, NGLs prepared from reducing oligosaccharides by oxime ligation with an aminooxy-functionalized DHPE (2); Cer, natural glycolipids with various ceramide moieties; GSC,designations for synthetic glycolipids in Kiso laboratory; Cer36, synthetic glycolipids with ceramide having a total of 36 carbon atoms; C30, a synthetic lipid [2-(tetradecyl)hexadecanol] with 30 carbon atoms. NB, Neu denotes de-N-acetylated neuraminic acid.

[b] Numerical scores for the binding signals are shown as means of duplicate spots at 5 fmol/spot (as in Figure 1) and are representative of at least 3 independent experiments;
-,Signal less than 1. Shades in grey are signals arisen from artefacts on the array slides with big error bars in Figure 1.

[c] Major component.

1. Chai W, Stoll MS, Galustian C, Lawson AM, Feizi T (2003) Neoglycolipid technology - deciphering information content of glycome. Methods Enzymol 362, 160-195.

2. Liu Y, et al. (2007) Neoglycolipid probes prepared via oxime ligation for microarray analysis of oligosaccharide-protein interactions. Chem Biol 14, 847-859.

Table S3 The relative real space correlation coefficient of the ligand to Trp149

	Trp149	SA-1	GAL-2
rVN1194 H5-LSTa	0.19(100)	0.16(84)	0.14(74)
rVN1194 H5-LSTc	0.17(100)	0.12(71)	0.11 (65) -l
rVN1194(Ala134Val) H5-LSTa	0.22(100)	0.09(41)	0.09(41)-p
rVN1194(Ala134Val) H5-LSTc	0.23(100)	0.08(35)	
rVN1194(Glu186Asp) H5-LSTa	0.20(100)	0.09(45)	0.06(30)
rVN1194(Glu186Asp) H5-LSTc	0.21(100)	0.1(48)	0.12(57)-p

-l: only the linker between SA and GAL-2

-p: partial of the GAL-2

Table S4 Data collection and refinement statistics of rVN1194 H5 HA

	H5-LSTa	H5-LSTc
**PDB number**	3ZP0	3ZP1
**Data collection**		
Space group	H32	H32
Cell dimensions		
a, b, c (Å)	101.2 101.2 449.8	101.1 101.1 448.6
α, β, γ (°)	90.0 90.0 120.0	90.0 90.0 120.0
Resolution (Å)	30.0-2.5(2.61-2.50)	30.0-2.6(2.72-2.60)
R_{merge}	7.2(41.9)	9.0(52.2)
$I/\sigma I$	25.2(3.0)	18.2(2.3)
Completeness (%)	94.0(64.8)	99.6(97.0)
Redundancy	14.9(9.5)	10.4(6.8)
**Refinement**		
Resolution (Å)	30.0-2.50	30.0-2.60
No. reflections	27704	24984
$R_{work/}$ R_{free}	18.2/23.6	22.5/27.2
No. atoms		
Protein	3821	3805
Ligand/ion	129	124
Water	213	116
B-factors		
Protein	65.5	64.2
Ligand/ion	110.0	116.6
Water	64.6	55.3
R.m.s deviations		
Bond lengths (Å)	0.009	0.009
Bond angles (º)	1.223	1.281

*Highest resolution shell is shown in parenthesis.

Table S5 Data collection and refinement statistics of rVN1194(Ala134Val) H5 HA mutant.

	H5(Ala134Val)-LSTa	H5(Ala134Val)-LSTc
**PDB number**	3ZP2	3ZP3
**Data collection**		
Space group	H32	H32
Cell dimensions		
a, *b*, *c* (Å)	101.2 101.2 449.3	101.1 101.1 449.2
α, β, γ (°)	90.0 90.0 120.0	90.0 90.0 120.0
Resolution (Å)	30.0-2.5(2.61-2.50)	30.0-2.65(2.77-2.65)
R_{merge}	7.6(55.1)	9.0(51.1)
$I/\sigma I$	19.6(3.8)	17.6(4.4)
Completeness (%)	100.0(100.0)	100.0(100.0)
Redundancy	10.4(10.1)	10.1(9.6)
**Refinement**		
Resolution (Å)	30.0-2.5	30.0-2.65
No. reflections	31234	26200
$R_{work/}$ R_{free}	22.1/27.6	22.9/26.9
No. atoms		
Protein	3842	3842
Ligand/ion	98/32	98/21
Water	207	77
B-factors		
Protein	59.0	54.3
Ligand/ion	107.7/128.4	106.0/128.4
Water	57.4	51.6
R.m.s deviations		
Bond lengths (Å)	0.010	0.009
Bond angles (º)	1.273	1.192

*Highest resolution shell is shown in parenthesis.

Table S6 Data collection and refinement statistics of rVN1194(Glu186Asp) H5 HA mutant

	H5 (Glu186Asp) -LSTa	H5(Glu186Asp) -LSTc
**PDB number**	3ZPb	3ZP6
**Data collection**		
Space group	H32	H32
Cell dimensions		
a, *b*, *c* (Å)	101.4 101.4 449.6	101.4 101.4 450.5
α, β, γ (°)	90.0 90.0 120.0	90.0 90.0 120.0
Resolution (Å)	30.0-2.30(2.40-2.30)	30.0-2.60(2.72-2.60)
R_{merge}	6.8(26.7)	7.8(51.4)
$I/\sigma I$	24.5(4.1)	21.8(4.3)
Completeness (%)	99.3(96.5)	99.1(97.3)
Redundancy	9.6(4.6)	10.3(8.2)
**Refinement**		
Resolution (Å)	30.0-2.30	30.0-2.60
No. reflections	39971	27780
R_{work}/R_{free}	21.8/26.0	22.3/26.0
No. atoms		
Protein	3839	3839
Ligand/ion	45/98	32/98
Water	167	154
B-factors		
Protein	55.2	70.6
Ligand/ion	123.1/109.9	135.8/125.3
Water	54.2	73.5
R.m.s deviations		
Bond lengths (Å)	0.008	0.009
Bond angles (º)	1.150	1.143

*Highest resolution shell is shown in parenthesis.

Table S7 Data collection and refinement statistics of rVN1194(Ile151Phe) H5 HA mutant

	H5(Ile151Phe)
**PDB number**	3ZPa
**Data collection**	
Space group	H32
Cell dimensions	
a, *b*, *c* (Å)	101.0 101.0 448.2
α, β, γ (°)	90.0 90.0 120.0
Resolution (Å)	30.0-2.50(2.61-2.50)
R_{merge}	9.0(66.7)
$I/\sigma I$	20.3(2.6)
Completeness (%)	98.3(97.9)
Redundancy	10.1(6.6)
**Refinement**	
Resolution (Å)	30.0-2.50
No. reflections	27673
$R_{work/}$ R_{free}	24.6/28.6
No. atoms	4019
Protein	3845
Ligand/ion	98
Water	76
B-factors	
Protein	41.0
Ligand/ion	137.4
Water	39.8
R.m.s deviations	
Bond lengths (Å)	0.008
Bond angles (º)	1.152
